# Flunarizine as a Candidate for Drug Repurposing Against Human Pathogenic Mammarenaviruses

**DOI:** 10.3390/v17010117

**Published:** 2025-01-16

**Authors:** Chukwudi A. Ofodile, Ikemefuna C. Uzochukwu, Fortunatus C. Ezebuo, InnocentMary Ejiofor, Mercy Adebola, Innocent Okpoli, Beatrice Cubitt, Haydar Witwit, Chetachi B. Okwuanaso, Ngozi Onyemelukwe, Juan Carlos de la Torre

**Affiliations:** 1 Department of Medical Laboratory Science, Faculty of Health Sciences and Technology, Nnamdi Azikiwe University, Awka 420218, Anambra, Nigeria; ac.ofodile@unizik.edu.ng (C.A.O.); cb.okwuanaso@unizik.edu.ng (C.B.O.); 2 Department of Immunology and Microbiology, Scripps Research Institute, La Jolla, CA 92037, USA; bcubitt@scripps.edu (B.C.); hwitwit@scripps.edu (H.W.); 3 Department of Medical Laboratory Sciences, Faculty of Health Sciences and Technology, University of Nigeria, Nsukka 401105, Enugu, Nigeria; ng_onyemelukwe@yahoo.com; 4 Department of Pharmaceutical & Medicinal Chemistry, Faculty of Pharmaceutical Sciences, Nnamdi Azikiwe University, Awka 420218, Anambra, Nigeria; ic.uzochukwu@unizik.edu.ng (I.C.U.); fc.ezebuo@unizik.edu.ng (F.C.E.); ni.okpoli@unizik.edu.ng (I.O.); 5 Department of Biochemistry, Graduate Center, City University of New York (CUNY), New York, NY 10016, USA; 6 Department of Pharmacognosy & Traditional Medicine, Faculty of Pharmaceutical Sciences, Nnamdi Azikiwe University, Awka 420218, Anambra, Nigeria

**Keywords:** Lassa virus, LCMV, minigenome, in silico docking, flunarizine, antiviral

## Abstract

Lassa fever (LF), a viral hemorrhagic fever disease with a case fatality rate that can be over 20% among hospitalized LF patients, is endemic to many West African countries. Currently, no vaccines or therapies are specifically licensed to prevent or treat LF, hence the significance of developing therapeutics against the mammarenavirus Lassa virus (LASV), the causative agent of LF. We used in silico docking approaches to investigate the binding affinities of 2015 existing drugs to LASV proteins known to play critical roles in the formation and activity of the virus ribonucleoprotein complex (vRNP) responsible for directing replication and transcription of the viral genome. Validation of docking protocols were achieved with reference inhibitors of the respective targets. Our in silico docking screen identified five drugs (dexamethasone, tadalafil, mefloquine, ergocalciferol, and flunarizine) with strong predicted binding affinity to LASV proteins involved in the formation of the vRNP. We used cell-based functional assays to evaluate the antiviral activity of the five selected drugs. We found that flunarizine, a calcium-entry blocker, inhibited the vRNP activity of LASV and LCMV and virus surface glycoprotein fusion activity required for mammarenavirus cell entry. Consistently with these findings, flunarizine significantly reduced peak titers of LCMV in a multi-step growth kinetics assay in human A549 cells. Flunarizine is being used in several countries worldwide to treat vertigo and migraine, supporting the interest in exploring its repurposing as a candidate drug to treat LASV infections.

## 1. Introduction

Lassa virus (LASV), the causative agent of Lassa fever (LF), is endemic to large regions of West Africa, where it is estimated to infect several hundred thousand people, resulting in many cases of LF, a febrile disease associated with high morbidity and case-fatality rates (CFRs) of 15–20% among hospitalized LF patients [1,2,3,4,5]. Since 2018, there has been an unprecedented rise in the incidence of Lassa fever (LF) cases in West African countries, including Nigeria. The CFR in confirmed cases of LF in Nigeria by November 2019 had reached 21%. LF has been listed as one of the priority diseases for research and countermeasures development by the World Health Organization (WHO) in 2018 [6,7,8]. LF is a hemorrhagic fever disease with epidemic potential and for which no licensed vaccines or specific therapies are currently available. The off-label use of the nucleoside analogue ribavirin (RIB) has been reported to provide clinical benefits if treatment is initiated within six days of onset of symptoms. However, the efficacy of RIB remains controversial [9,10] and it can cause significant side effects [11,12]. Hence, the development of therapeutics to treat LF represents an unmet problem of clinical significance.

LASV is an enveloped virus with a bi-segmented negative-stranded RNA genome [13,14]. Each genome segment uses an ambisense coding strategy to express two viral proteins from open reading frames separated by non-coding intergenic regions (IGRs). The large (L) segment encodes the virus RNA-dependent RNA polymerase, L protein, and the matrix Z protein, whereas the small (S) segment encodes the nucleoprotein (NP) and the glycoprotein precursor (GPC) [13,15,16]. GPC is co-translationally processed by cellular signal peptidase to generate a stable signal peptide (SSP) and a GPC precursor that is post-translationally cleaved by the cellular proprotein convertase subtilisin kexin isozyme-1/site-1 protease (SKI-1/S1P) to generate GP1 and GP2 subunits. The GP1 and GP2 subunits, together with the SSP, form mature glycoprotein (GP) peplomers on the surface of the virion envelope that mediate virion cell entry via receptor-mediated endocytosis. LASV GP1 interacts with α-dystroglycan [17,18] and the lysosomal-associated membrane protein 1 (LAMP-1) [19] to facilitate virus cell entry. GP2 is responsible for mediating the pH-dependent fusion of the virus and the host cell membranes to complete the entry process [20,21]. NP is the most abundant viral protein in virions and infected cells. As with other mammarenaviruses, LASV NP encapsidates the viral genome to generate the nucleocapsid (NC) template that—in association with the virus RdRp L protein—forms the viral ribonucleoprotein complex (vRNP) responsible for directing the biosynthetic processes of replication and transcription of the viral genome [13]. The C-terminal region of NP contains a 3′-5′ exoribonuclease domain (ExoN) of type DEDDH that has been implicated in NP’s anti-interferon activity and viral fitness [22], as well as evading PKR kinase activity [23]. The LASV matrix Z protein, a myristoylated protein, plays critical roles in the assembly and cell egress via budding of viral particles [24,25], a process that can be targeted using N-myristoyl transferase inhibitors [26].

Several experimental drugs targeting different steps of the LASV life cycle are currently being investigated, or repurposed, as candidate therapeutics to treat mammarenavirus, including LASV infections [27], and the LASV GP-mediated fusion inhibitor LHF-535 is in phase I clinical trials [28]. However, the uncertainty about their in vivo efficacy underscores the need for research aimed at expanding the number and diversity of candidate drugs to treat infections by human pathogenic mammarenaviruses, including LASV. In the present work, using in silico docking simulation methods, we identified five existing drugs (dexamethasone, ergocalciferol, flunarizine, mefloquine, and tadalafil) with predicted high binding affinities to the endonuclease (EndoN) domain located at the N-terminal region of LASV L, the ExoN domain of NP and the Z protein. We tested their antiviral activity using LASV and LCMV cell-based minigenome systems and LCMV cell-based infection assays. We found that flunarizine (FLN) inhibited the activity of LASV and LCMV MG, a surrogate of vRNP activity, and significantly reduced (≥1 log) peak titers of LCMV in a multi-step growth kinetics assay in human A549 cells. In addition to its effect on vRNP activity and unanticipated based on the in silico docking results, FLN inhibited GP2-mediated fusion required for completion of virus cell entry. Our findings are consistent with the activity of FLN as a selective calcium-entry blocker antagonist [29,30], as Ca^2+^ flow and signaling have been shown to contribute to different steps of multiplication of different viruses [31,32,33,34]. However, other selected calcium channel blockers, including the combined L-/T-type (verapamil and nickel chloride) [35,36,37,38] and L-type (nifedipine and gabapentin) [39,40,41,42] calcium channel blockers, did not exhibit significant anti-LCMV activity, suggesting that FLN anti-mammarenaviral activity may not be related to its ability to block T-type calcium channels. FLN binds calmodulin, which can interfere with the role of calmodulin in low-pH-induced intracellular membrane fusion [43], thus interfering with GP2-mediated, pH-induced fusion required for mammarenavirus cell entry.

FLN is a selective calcium-entry blocker antagonist that is being used in many countries worldwide to treat vertigo and migraine, supporting the interest in exploring its repurposing as a candidate drug to treat LASV infections.

## 2. Materials and Methods

### 2.1. In Silico Screening

The 3D structures of LASV L polymerase (PDB, 5J1N), NP (PDB, 3q7c), GP1 (PDB, 4ZJF), GP2 (PDB, 5OMI), and Z matrix protein (PDB, 2M1S) were obtained from the Protein Data Bank (PDB), examined with PyMol-1.4.1, and appropriately prepared for molecular docking simulations using Chimera-1.9 [44] and MGLTools-1.5.6 [45]. A total of 2015 existing drugs were obtained from DrugBank and prepared for molecular docking simulations using MGLTools-1.5.6 [45]. Briefly, all hydrogen and Gasteiger charges were added, roots were detected, and torsions and all rotatable bonds were allowed in their natural states. Then, outputs were generated as a PDBQT file extension and used for the virtual screening after validation of docking protocols.

Before molecular docking simulations, the reference compounds and their respective targets were subjected to blind docking and re-docking. Briefly, the reference compounds and all hetero-molecules in the drug target were deleted using Chimera-1.9 [44]. Polar hydrogen, Kollman charges, grid box sizes and centers (Table 1) at grid spacing of 1.0 Å were determined with MGLTools-1.5.6 [45]. The reference compound was subjected to 1000 steps of steepest descent and 100 steps of conjugate gradient energy minimization at a step size of 0.02 using Chimera-1.9. Then, it was prepared for molecular docking simulations using MGLTools-1.5.6 [45]. All hydrogen and Gasteiger charges were added to the reference compounds and roots were detected. Then, torsions and all rotatable bonds were allowed in their natural states. Output was then generated as a PDBQT file extension. The targets and their respective reference compounds and the prepared existing drugs were used for the docking simulations/virtual screening. The docking simulations/virtual screening were performed with AutoDock Vina [46] using the parameters presented in Table 1.

The compound library set was batched for molecular docking simulations against the three LASV protein targets using virtual screening scripts. Molecular docking simulations were implemented, with four replicates, on a Linux platform using AutoDock Vina [46] and associated tools after validation of docking protocols. For The L protein, docking was performed using the endonuclease (EndoN) domain of LASV L (PDB ID: 5J1N). Blind docking was initially performed to identify the favorable binding site/pocket for the reference compound (efavirenz). Thereafter, refined docking simulation was performed using the docking parameters in Table 1. The NP docking was performed using the 3′-5′ exonuclease (ExoN) domain of LASV NP (PDB ID: 3Q7C). Blind docking was initially performed to identify the favorable binding site/pocket for the reference compound (Fmoc_D_Cha_OH). Thereafter, refined docking simulation was performed using the docking parameters in Table 1. Initial inspection of the Z protein (PDB ID: 2M1S) showed it had some binding pockets. Blind docking was initially performed to identify the favorable binding site/pocket for the reference compound (acyclovir). Thereafter, refined docking simulation was performed using the docking parameters in Table 1. Binding free energy values (kcal/mol ± SD) were ranked to identify the front-runner compounds. The inhibition constant (Ki) was obtained from the binding energy (ΔG) using the formula Ki = exp(ΔG/RT), where R is the universal gas constant (1.985 × 10^−3^ kcal mol^−1^ K^−1^) and T is the temperature (298.15 K). Existing drugs with strong L, NP, and Z binding affinities were selected as potential candidate therapeutics for the treatment of LF. Molecular descriptors of selected compounds were extracted from relevant databases.

To select existing drugs for possible treatment of mild infections of Lassa fever (early presenters), we first sorted existing drugs with very strong polymerase binding affinities (with efavirenz as cutoff compound), then sorted the resulting list for good myristoylation binding affinities (with acyclovir as cutoff compound) before finally sorting the list for good nuclease binding affinities (with FMOC_D_Cha_OH as cutoff compound). Similarly, existing drugs with very strong nuclease binding affinities (with FMOC_D_Cha_OH as cutoff compound) were first sorted from the list. The resulting list was subsequently sorted for good myristoylation binding affinities (with lassamycin as cutoff compound), and finally the remaining existing drugs were sorted for good polymerase binding affinities (without any cutoff compound) to generate the list of drugs for possible treatment of severe infections of Lassa fever (late presenters). The LF virus protein residues responsible for interactions with some existing front-runner drugs were also explored in the respective receptor–ligand complexes using Discovery Studio 2024.

### 2.2. Source of Compounds

Flunarizine (FLN) dihydrochloride (HY-B0358A, MedChemExpress, Monmouth Junction, NJ, USA), mefloquine (MEF) hydrochloride (HY-17437A MedChemExpress, NJ, USA), ergocalciferol (vitamin D) (HY-76542 MedChemExpress, NJ, USA), tadalafil (TAD) (HY-90009A MedChemExpress, NJ, USA), dexamethasone (DEX) (D2915 Sigma-Aldrich, St. Louis, MO, USA).

### 2.3. Cells and Viruses

HEK 293T (ATCC CRL-3216), Vero E6 (ATCC CRL-1586), and A549 (ATCC CCL-185) cell lines were maintained in Dulbecco’s modified Eagle’s medium (DMEM; Thermo Fisher Scientific, Vacaville, CA, USA) supplemented with 10% heat-inactivated fetal bovine serum (FBS), 2 mM L-glutamine, 100 µg/mL streptomycin, and 100 U/mL of penicillin. Recombinant viruses rLCMV/GFP-P2A-NP [47], r3JUNVCandid GFP/GFP [48], rARMΔGPC/ZsG-P2A-NP [23,49] have been described.

### 2.4. Virus Titration

Virus titers were determined by focus-forming assay (FFA) [50] using Vero E6 cells. Briefly, cells were seeded in a 96-well plate (2 × 10^4^ cells/well) and the next day infected with 10-fold serial virus dilutions. At 20 h post infection (hpi), cells were fixed with 4% paraformaldehyde (PFA) in PBS and infected cells were identified by epifluorescence based on their GFP expression.

### 2.5. Cell-Based Minigenome (MG) Assays

The LASV and LCMV MG systems have been described [51,52], and these were used to assess the anti-vRNP activity of hits identified by in silico docking.

#### 2.5.1. LASV MG Assay

HEK293T cells were seeded at 3.0 × 10^5^ cells per well onto a poly-L-lysine treated 12-well plate 20 h prior transfection with pCAGGS-T7 Cyt (0.5 μg), pT7MG-ZsGreen (0.5 μg), pCAGGS-NP (0.3 μg), and pCAGGS-L (0.6 μg) plasmids using Lipofectamine 2000 (2.5 μL/μg of DNA). After 5 h, the transfection medium was exchanged with fresh medium containing 20 µM of test compounds. Cells were lysed 48 h after transfection and ZsGreen expression levels were measured. Briefly, whole-cell lysates were prepared using 0.2 mL of buffer (50 mM Tris pH 7.4, 1 mM EDTA, 0.5% NP-40, and 150 mM NaCl). ZsGreen expression levels in clarified lysates were quantified using a Synergy H4 Hybrid MultiMode microplate reader (BioTek Instruments, Winooski, VT, USA). The measurements were normalized to total cell protein content, determined using a Pierce BCA Protein Assay Kit (Thermo Fisher Scientific). The mean values were then normalized to the vehicle (DMSO)-treated control, which was set to 100%. LASV L and NP sequences used in the MG assay were those from the Josiah strain.

#### 2.5.2. LCMV MG Assay

HEK293T cells were plated at a density of 3.0 × 10^5^ cells per well onto a poly-L-lysine-treated 12-well plate 20 h prior to transfection with pCAGGS-T7 Cyt (0.5 μg), pT7MG-GFP (0.5 μg), pCAGGS-NP (0.3 μg), and pCAGGS-L (0.6 μg) plasmids using Lipofectamine 2000 (2.5 μL/μg of DNA). After 5 h, the transfection medium was exchanged with fresh medium containing 20 µM of test compounds. At 48 h post-transfection, whole-cell lysates were prepared using 0.2 mL of buffer (50 mM Tris pH 7.4, 1 mM EDTA, 0.5% NP-40, and 150 mM NaCl) and GFP expression levels in clarified lysates quantified using the Synergy H4 Hybrid MultiMode microplate reader (BioTek Instruments, Winooski, VT, USA). The measurements were normalized to total cell protein content, determined using the Pierce BCA Protein Assay Kit (Thermo Scientific). The mean values were then normalized to the vehicle (DMSO)-treated control, which was set to 100%.

### 2.6. Cell Viability Assay

CellTiter 96 AQueous One Solution reagent (Promega, CAT#: G3580, Madison, WI, USA) was utilized to evaluate cell viability. This assay quantifies the number of living cells by measuring the amount of formazan produced from MTS [3-(4,5-dimethylthazol-2-yl)-5-(3-carboxymethoxyphenyl)-2-(4-sulfophenyl)-2H-tetrazolim] through the action of NADPH or NADH generated in viable cells. Cells were plated on a 96-well optical plate at 3.0 × 10^4^ cells/well. After 20 h of incubation, compounds (at indicated concentrations in four replicates) or vehicle control were added to the cells in a final volume of 100 µL. At 48 h post-treatment, CellTiter 96 AQueous One solution reagent (Promega) was added to each well, and the plate was incubated for 15 min at 37 °C/5% CO_2_. Absorbance measurements were taken at 490 nm using an enzyme-linked immunosorbent assay (ELISA) reader (SPECTRA max plus 384, Molecular Devices, Sunnyvale, CA, USA). The results were normalized to the vehicle control group (DMSO), which was assigned a value of 100%.

### 2.7. Determination of Compounds EC_50_ and CC_50_ in an LCMV Cell-Based Infection Assay

Cells were seeded onto a 96-well optical plate at 3.0 × 10^4^ cells/well and incubated for 16 h at 37 °C and 5% CO_2_. Cells were infected with rLCMV/GFP-P2A-NP at an MOI of 0.03 and treated with 3-fold serial dilutions of the indicated compound starting at 100 µM (four replicates for each compound and dilution concentration) for 48 h prior to CC_50_ and EC_50_ determination. CellTiter 96 Aqueous One Solution was used to determine the CC_50_ with values normalized to vehicle-treated control (0.2% DMSO) that was adjusted to 100%. To determine EC_50_ values, the CellTiter 96 Aqueous One Solution and media were aspirated and the cells fixed with 4% paraformaldehyde. GFP expression levels were determined by fluorescence using a Biotek Cytation 5 plate reader (Agilent Technologies, Santa Clara, CA, USA). Mean values were normalized to infected and vehicle (DMSO)-treated control that was adjusted to 100%. EC_50_ and CC_50_ values were determined using GraphPad Prism v10 (GraphPad Software, San Diego, CA, USA).

### 2.8. Virus Multi-Step Growth Kinetics

A549 cells were seeded at 2.0 × 10^5^ cells per well in a 12-well plate and the next day infected with rLCMV/GFP-P2A-NP an MOI of 0.03. After 90 min adsorption, the inoculum was removed and cells treated with VC (0.5% DMSO) or FLN (50 µM) or RIB (100 µM) as a positive control. Tissue culture supernatants (TCS) were collected at 24, 48, and 72 hpi. At the experimental end point (72 hpi) cells were stained with Hoechst dye solution. Live cell images were captured using a Keyence BZ-X710 all-in-one fluorescence microscope series. Cells were washed and RNA isolated using TRI Reagent (TR 118, Molecular Research Centre, Cincinnati, OH, USA). The titers of the TCS were determined by FFA using Vero E6 cells (four replicates), with values presented as mean ± SD. Data were plotted using GraphPad Prism (GraphPad Software, San Diego, CA, USA).

### 2.9. RT-qPCR

RT-qPCR was performed as described in [23]. RNA was isolated using TRI Reagent according to the manufacturer’s instructions. The isolated RNA was resuspended in RNA storage solution (Life Technologies, Carlsbad, CA, USA. AM 7000) and quantified using a NanoDrop™ 2000 spectrophotometer (ND-2000 Thermo Fisher Scientific™). RNA (1 µg) was reverse-transcribed to cDNA using the SuperScript IV first-strand synthesis system (18091050, Life Technologies). Powerup SYBR (A25742, Life Technologies) was used to amplify LCMV NP, and the housekeeping gene *GAPDH* using the following primers: NP forward (F): 5′ CAGAAATGTTGATGCTGGACTGC-3′ and NP reverse (R): 5′-CAGACCTTGGCTTGCTTTACACAG-3′; *GAPDH* F: 5′-CATGAGAAGTATGACAACAGCC-3′ and GAPDH R: 5′-TGAGTCCTTCCACGATACC-3′.

### 2.10. Time of Addition Assay

A549 cells were seeded at 3 × 10^4^ cells per 96-well (black-walled optical) plate (353219, Falcon) and grown for 16 h at 37 °C and 5% CO_2_. Cells were treated with VC (0.25% DMSO), FLN (50 µM), and F3406 (5 µM) (four replicates for each treatment) starting 2 h prior infection or 2 h post-infection with the single-cycle infectious rARMΔGPC/ZsG-P2A-NP (MOI of 1.0), which eliminated the need for NH_4_Cl treatment to prevent the confounding factor introduced by multiple rounds of infection. At 24 hpi, cells were fixed with 4% PFA and ZsGreen expression levels were measured using a Cytation 5 imaging reader.

### 2.11. Budding Assay

Budding assays using the Z-Gaussia luciferase-based assay (LASV-Z-GLuc plasmid) [53] were performed. Briefly, 293T cells (1.75 × 10^5^ cells/well in 12-well plate) were transfected with 0.5 μg of pC.LASV-Z_Gluc or pC.LASV-Z-G2A-GLuc (mutant control) or pCAGGS-Empty (pC-E) using Lipofectamine 2000. After 5 h transfection, cells were washed three times and treated with FLN (50 µM), RIB (100 µM), or vehicle control (VC). After 48 h of treatment, cell culture supernatants (CCSs) containing virus-like particles (VLPs) and cells were collected. CCS samples were clarified from cell debris by centrifugation (13,000 rpm/4 °C/10 min) and aliquots (20 µL each) from CCS samples were added to 96-well black plates (VWR, West Chester, PA, USA) and 50 µL of Steady-Glo luciferase reagent (Promega) added to each well. Cell lysates were prepared using 250 µL of lysis buffer (1% NP-40, 50 mM Tris-HCl (pH 8.0), 62.5 mM EDTA, 0.4% sodium deoxycholate). Lysates were clarified from cell debris by centrifugation (13,000 rpm/4 °C/10 min). GLuc activity in Z-containing VLP and whole-cell lysates (WCLs) was determined using the Steady-Glo luciferase assay system (Promega, Madison, WI, USA) according to the manufacturer’s protocol using a Berthold Centro LB 960 luminometer (Berthold Technologies, Oak Ridge, TN, USA). The GLuc activity in CCS and WCL served as a surrogate for Z protein levels. Z budding efficiency (in %) was determined by the ratio of VLP-associated GLuc levels (Z_VLP_) and total GLuc levels (Z_VLP_ + Z_WCL_) times 100.

### 2.12. GPC-Mediated Cell Fusion Assay

HEK293T cells were seeded onto poly-L-lysine coated wells in a 24-well plate (1.25 × 10^5^ cells/well). Following an incubation period of 20 h, cells were transfected with plasmids expressing LCMV GPC together with a plasmid expressing GFP or with a plasmid expressing GFP alone using Lipofectamine 3000. At 18 h post-transfection, the transfection mixture was removed and replaced with DMEM containing 10% FBS and FLN (50 µM) or VC. At 5 h post-treatment, 0.5 mL of acidic (pH 5.0) or neutral (pH 7.2) DMEM was added to the cells and incubated for 15 min (37 °C/5% CO_2_), washed once with DMEM +10% FBS, and 0.5 mL of DMEM containing 10% FBS and FLN (50 µM) or VC was added per well. Cells were fixed after 2 h with 4% PFA and stained with DAPI. Syncytium formation was visualized and imaged using a Keyence BZ-X710 microscope to record the GFP expression.

### 2.13. Virucidal Assay

rLCMV/GFP-P2A-NP (10^5^ FFU) was treated with FLN at 1, 50, and 100 µM or VC for 30 min at room temperature. The infectivity of the virus after treatment was determined by FFA using Vero E6 cells. Infected cells were identified based on their GFP expression and are presented as means ± SD (four replicates). The counts were normalized (%) to vehicle-treated infected cells, which was set at 100%.

### 2.14. Epifluorescence

Images were collected using the Keyence BZ-X710 microscope. Images were transferred to a laptop for data processing purposes. Microsoft PowerPoint 2019 was used to assemble and arrange the images, with each one being imported separately and arranged in a cohesive manner within its respective composite. The canvas size was adjusted to ensure a harmonious layout.

### 2.15. Statistical Analysis

All statistical analyses were conducted using GraphPad Prism software v.10.2.3 (403) (GraphPad).

## 3. Results

### 3.1. In Silico Docking Screen to Identify Candidate Drug Inhibitors of LASV Multiplication

**Figure 1:**
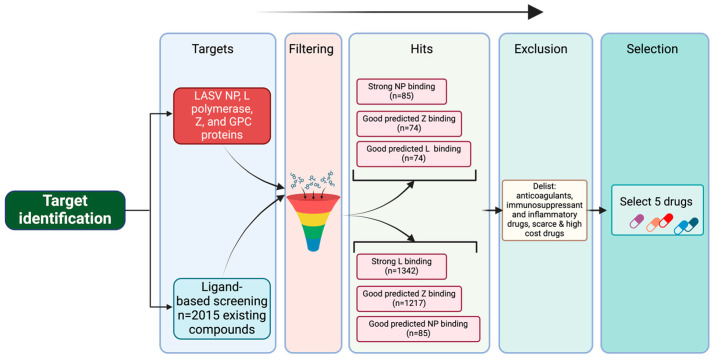
In silico docking flowchart. To identify existing drugs that could be potentially repurposed to treat cases of LF, we performed an in silico docking screen of 2015 existing drugs from DrugBank to identify those predicted to target LASV proteins and their activities (Figure 1). We specifically focused on candidate drugs targeting activities associated with LASV L, NP and Z proteins. Our in silico screening identified five drugs with high predicted binding affinities to one or more of the LASV target proteins (Table 2). Amino acid residues in LASV proteins predicted to be responsible for interactions with the selected drugs were explored in the respective receptor–ligand complexes using Discovery Studio 2024 (Table 3).

To identify potential anti-LASV compounds, we conducted an in silico screen of 2015 approved drugs potentially binding to LASV proteins (L, Z, NP, and GPC) (Figure 1). Based on docking parameters, we did not pursue potential GPC interactors (Supplementary Table S1). Our in silico docking screen identified five (5) approved drugs with favorable docking scores (Table 1 and Table 2). The five approved drugs were further subjected to in vitro validations.

### 3.2. Effect of Selected Hits on the Activity of LASV and LCMV vRNPs

**Figure 2:**
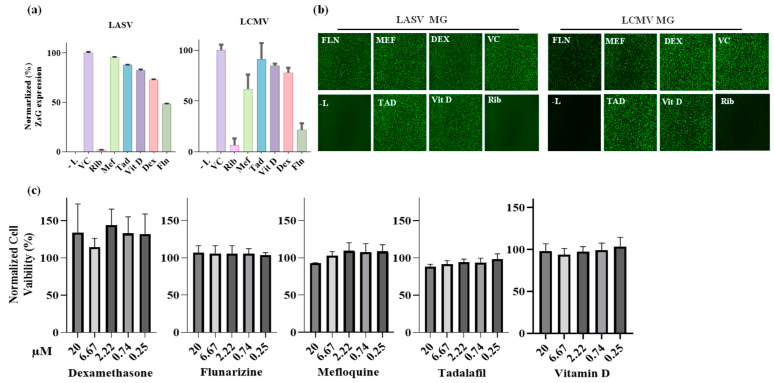
Effect of selected compounds on the LASV and LCMV vRNP activity in cell-based MG assays. (**a**) Compound effect on MG-directed ZsG (LASV-MG) or GFP (LCMV-MG) expression. HEK293T cells were seeded in 12-well plate (3.0 × 10^5^ cells/well) and 16 h later overnight transfected with plasmids (pCAGGS) expressing LASV or LCMV trans-acting factors NP and L and a plasmid that allowed for intracellular T7 RNA polymerase mediate synthesis of the LASV or LCMV MG RNAs. At 5 h post transfection, the transfecting medium was replaced with media containing the indicated compounds at 20 µM. At 48 h post-transfection, whole-cell lysates were harvested to determine levels of ZsGreen (LASV-MG) or GFP (LCMV MG). ZsGreen and GFP measurements were normalized to total protein in lysate and all samples were normalized to vehicle control. The experiment was conducted in four replicates, and indicated values represent the mean ± standard deviation (SD). (**b**) Epifluorescence images of cells expressing LASV-MG (ZsG) or LCMV-MG (GFP) in the presence of the indicated compounds were collected after fixing with 4% PFA at 48 h post-transfection using the Keyence BZ-X710 imaging system. (**c**) Compound effect on cell viability. The dose-dependent effect of the indicated compounds on HEK293T cell viability was determined using a CellTiter 96^®^ AQueous One Solution cell proliferation assay. Results correspond to the means ± standard deviation (SD) of four biological replicates.

Based on the parameters of the in silico docking screen, we selected MEF, TAD, DEX, Vit D, and FLN as the top candidate hits for testing their ability to inhibit the activity of LASV and the closely related mammarenavirus LCMV vRNPs. For this, we used described LASV and LCMV cell-based minigenome (MG) assays [54]. These MG systems recapitulate the steps involved in LASV and LCMV RNA synthesis using an intracellular reconstituted vRNP that directs expression of a reporter gene (ZsGreen for LASV-MG and GFP for LCMV-MG) whose expression levels serve as an accurate surrogate of the vRNP activity. Intracellular reconstitution of LASV and LCMV vRNP requires co-expression of the corresponding viral L and NP proteins, as well as a plasmid to launch intracellular synthesis of the viral MG vRNA. In these experiments, expression levels of the MG reporter serve as a comprehensive measurement of LASV and LCMV MG replication, transcription, and translation of the MG-encoded reporter gene. We first assessed the effect of each compound on the viability of HEK293T cells and found that none of the five tested compounds had noticeable effects on cell viability when used up to 20 µM, the concentration we selected for the cell-based MG assay (Figure 2c). We then transfected HEK293T cells with the components of the LASV or LCMV MG system and treated them with the indicated compounds (20 µM), vehicle control (VC), or RIB (100 µM), a validated inhibitor of LASV and LCMV replication (Figure 2a). At 48 h post-transfection, whole-cell lysates were prepared and expression levels of ZsGreen (LASV-MG) and GFP (LCMV-MG) determined and normalized to the amount of total protein in the corresponding sample. Mean values (four replicates) of reporter gene expression were normalized by assigning a value of 100% to the vehicle control (VC)-treated sample. In parallel, cells transfected with the same combination of plasmids were fixed with 4% PFA at 48 h post-transfection and representative fields imaged using a Keyence BZ-X710 imaging system (Figure 2b). Compared to VC-treated cells, we observed a significant (~50%) reduction in LASV MG-directed ZsGreen expression levels in cells treated with FLN. We observed a much lower effect on LASV MG activity in cells treated with any of the other selected hits: dexamethasone (~28% reduction), VIT D (~18% reduction), TAD (~12% reduction), and MEF (5% reduction) (Figure 2a). We observed a similar pattern of compound-mediated inhibition of the LCMV MG activity, with FLN exhibiting the strongest inhibitory effect (Figure 2a).

### 3.3. Dose-Dependent Inhibitory Effect of Selected Drugs on LCMV Multiplication in Cultured Cells

Selected hits from the in silico docking screen exhibited similar activity patterns against LASV and LCMV vRNP, suggesting that these drugs may have a similar inhibitory effect on the multiplication of LASV and LCMV. We therefore used the BSL2 agent LCMV to test the compounds in a cell-based infection assay, avoiding the need for the highest BSL4 containment required for the use of live LASV [55].

**Figure 3:**
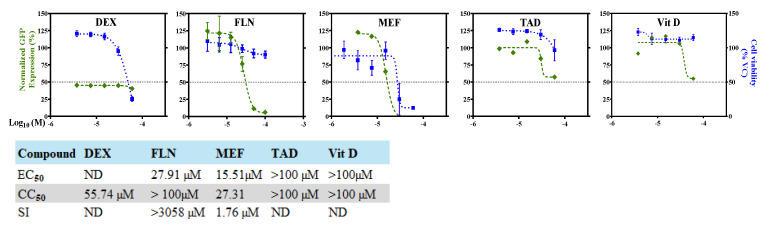
Dose–response curves of selected compounds on LCMV multiplication in Vero cells. Vero cells were seeded (3.0 × 10^4^ cells/well) in a 96-well plate and 16 h later infected with rLCMV/GFP-P2A-NP at an MOI of 0.03. After 90 min adsorption, the virus inoculum was removed and infected cells treated with the indicated compounds at different concentrations. At 48 h post-infection, cell viability was evaluated using CellTiter 96 Aqueous One solution reagent (Promega). The cells were washed and fixed, and GFP expression levels were measured using a fluorescence plate reader. The dose–response assay was performed in four replicates for each compound, and the mean values were normalized to the vehicle control. EC_50_, CC_50_, and SI values are shown. ND, not determined.

We determined the dose-dependent effect of MEF, TAD, DEX, Vit D, and FLN on rLCMV/GFP multiplication in A549 cells. For this, we infected A549 cells with rLCMV/GFP (MOI = 0.03) and treated them with threefold serial dilutions of each compound. At 48 h post-infection (hpi) CellTiter 96 AQueous One Solution Reagent was added to the cells, and after incubation for 35 min (37 °C and 5% CO_2_), absorbance values were collected. Cells were then fixed (4% PFA/PBS) and GFP expression levels measured using a fluorescence plate reader (Figure 3). Normalized cell viability values and GFP expression levels were used to determine drug CC_50_ and EC_50_ values using GraphPad Prism software v10 (Figure 3). Consistent with the cell-based MG assay results, FLN was the drug with the best antiviral profile in the LCMV cell-based infection assay, with EC_50_ of 27.91 µM and a CC_50_ of above 100 µM. MEF exhibited moderate anti-LCMV activity in Vero cells (EC_50_ of 15.51 µM), but due to toxicity (CC_50_ of 29.12 µM) showed a very low selective index (SI) of 1.87. DEX, Vit D and TAD had minimal toxicity on Vero cells and exhibited very poor inhibitory activity on LCMV multiplication.

### 3.4. Characterization of the Effect of FLN on LCMV Multi-Step Growth Kinetics in A549 Cells

**Figure 4:**
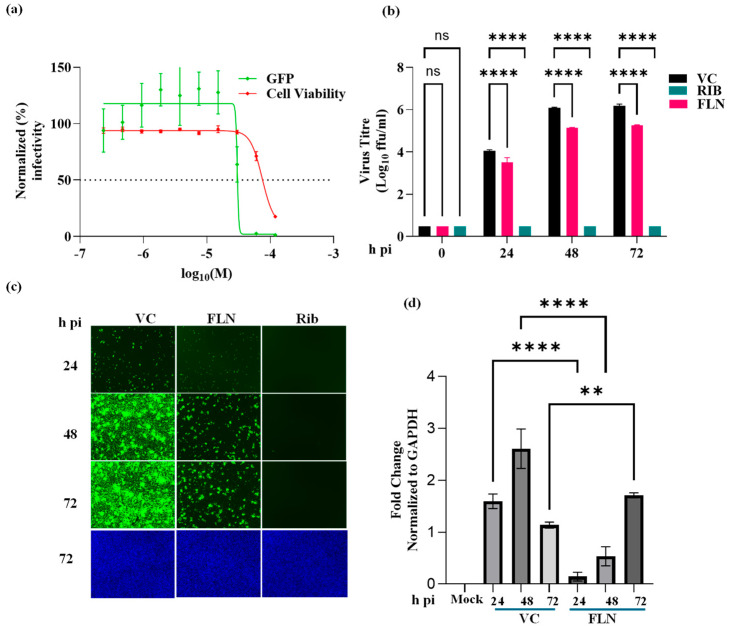
Effect of FLN on LCMV multiplication in human A549 cells. (**a**) Dose-dependent effect of FLN on LCMV multiplication. A549 cells were seeded (3.0 × 10^4^ cells/well) in a 96-well plate and 16 h later infected with rLCMV/GFP-P2A-NP at an MOI of 0.03. After 90 min adsorption, the virus inoculum was removed and infected cells treated with the indicated concentrations of FLN (four replicates per concentration). At 72 h post infection, cells were fixed and stained with DAPI. GFP expression levels and DAPI staining were used to determine EC_50_ and CC_50_, respectively. Values correspond to the means ± SD of four replicates. (**b**) Effect of FLN (50 µM) on LCMV multi-step growth kinetics and peak titers. A549 cells were seeded (2.0 × 10^5^ cells/well in 12-well plate) and 16 h later infected with rLCMV/GFP-P2A-NP at an MOI of 0.03. After 90 min adsorption, the virus inoculum was removed and infected cells treated with FLN (50 µM) or RIB (100 µM) as a control. At the indicated time points, post-infection tissue culture supernatants were collected and virus titers determined by FFA using Vero cells. Virus titers shown correspond to the means ± SD of six biological replicates. Two-way analysis of variance with Dunnett correction for multiple comparisons was implemented for statistical analysis. **** *p* < 0.0001. (**c**) Effect of FLN on virus cell propagation of LCMV. After collection of CCSs, cells were washed and subjected to Hoechst staining, and images were taken using Keyence BZ-X710 at ×4 magnification. (**d**) Effect of FLN on levels of viral RNA determined by RT-qPCR. After images of live cells were collected (**c**), cells were washed and total RNA isolated from each sample using TRI reagent, and RT-qPCR was used to determine levels of NP gene expression for each treatment normalized to GAPDH. Relative quantification was calculated using the untreated sample as the calibrator, and the means ± SD were plotted. One-way analysis of variance with Šidák correction for multiple comparisons was implemented for statistical analysis. ** *p* < 0.01, **** *p* < 0.0001. Statistical significance *p* < 0.05.

FLN exhibited the highest potency among all five compounds assessed, and we used Discovery Studio to identify the specific amino acid residues in each viral protein predicted to interact with FLN (Supplementary Figures S1 and S2). To further investigate the effect of FLN on mammarenavirus multiplication, we first examined the dose-dependent effect of FLN on LCMV multiplication (Figure 4a) in A549 cells.

A549 cells were seeded (3.0 × 10^4^ cells/well) in a 96-well plate and 16 h later infected with rLCMV/GFP-P2A-NP at an MOI of 0.03. After 90 min adsorption, the virus inoculum was removed and infected cells treated with the indicated concentrations (different doses) of FLN (four replicates per concentration). At 72 hpi, cells were fixed with 4% PFA and stained with DAPI. GFP expression levels and DAPI staining were used to determine virus infectivity and cell viability, respectively. Values correspond to the means ± SD of four replicates. Levels of GFP expression and DAPI staining were normalized, assigning a value of 100% to GFP expression levels and DAPI staining of VC-treated and infected cells. Normalized values were used to determine the EC_50_ and CC_50_. FLN had an EC_50_ of 30.11 µM, CC_50_ of 74.3 µMn, and SI of 2.5. We next examined the effect of FLN on the production of infectious viral progeny (Figure 4b) and virus cell propagation (Figure 4c) in A549 following infection with rLCMV/GFP-P2A-NP at an MOI of 0.03. We used treatment with RIB (100 µM) as a benchmark for a validated inhibitor of mammarenavirus multiplication. At the indicated hpi, cell culture supernatants (CCSs) were collected, the media replaced with FluoroBrite DMEM, and cells stained with Hoechst. Virus titers in CCS samples were determined using a FFA and Vero E6 as cell substrate (Figure 4b). Representative images of each sample were obtained using live-cell fluorescence microscopy (Figure 4c). After images were collected, total cellular RNA was isolated from each sample and levels of LCMV NP RNA determined by RT-qPCR (Figure 4d) for each treatment and normalized to levels of GAPDH. Relative quantification was performed using untreated samples as reference. Average values ± SD were plotted (*p* < 0.05). FLN (50 µM) caused a 1-log reduction in virus peak titers (Figure 4b) that correlated with reduced virus cell propagation (Figure 4c), as well as levels of viral RNA determined by RT-qPCR (Figure 4d).

### 3.5. Effect of FLN Treatment on Different Steps of LCMV Life Cycle

**Figure 5:**
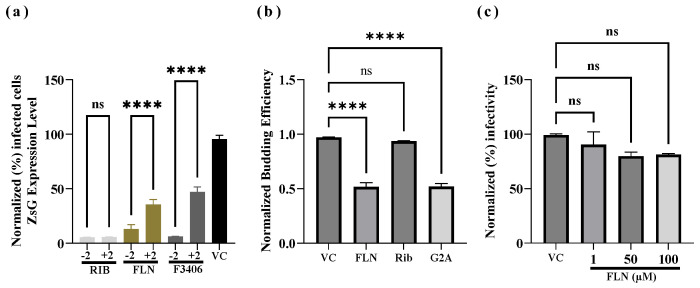
Effect of FLN on different steps of the LCMV life cycle. (**a**) Time of addition Assay. A549 cells were seeded in a 96-well plate (3.0 × 10^4^ cells/well). After overnight incubation, cells were treated with FLN (50 µM), VC, and the LCMV cell entry inhibitor F3406 (5 µM) starting 2 h prior (−2) or 2 h post (+2) infection with the single-cycle infectious rARMΔGPC/ZsG-P2A-NP at an MOI of 1.0. ZsGreen expression levels were determined at 48 h post-infection and mean values normalized to VC-treated wells set to 100%. Values of ZsG from −2 and +2 samples of each treatment were analyzed by ordinary one-way ANOVA using GraphPad Prism. **** *p* < 0.0001; ns indicates statistically not significant. (**b**) Budding assay. HEK293T cells were seeded (1.75 × 10^5^ cells/well) into poly-l-lysine-coated wells in a 12-well plate. The next day, cells were transfected with either pC.LASV-Z-GLuc, pC.LASV-Z-G2A-GLuc (mutant control), or pCAGGS-Empty (pC-E). After 5 h transfection, cells were washed three times and treated with FLN (50 µM), VC, and RIB (100 µM). At 48 h post-transfection, tissue culture supernatants (TCSs) were collected, the cells washed, and whole-cell lysis (WCL) collected. GLuc activity was measured in the TCS (Z_VLP_) and WCL. The WCL values were normalized with the total protein in the lysate (Z_WCL_) using the Steady-Glo Luciferase Pierce: Gaussia Luciferase Glow assay kit on a Cytation 5 reader. Budding efficiency, defined as Z_VLP_/(Z_VLP_ + Z_WCL_), was normalized, then plotted and analyzed (one-way ANOVA) using GraphPad Prism software (v.10). **** *p* < 0.0001; ns indicates statistically not significant. (**c**) Virucidal assay. rLCMV/GFP-P2A-NP (1 × 10^5^ FFU/mL) was treated with FLN at the indicated concentrations for 30 min at room temperature. The infectivity of the virus after treatment was determined by FFA using Vero E6 cells. Tenfold dilutions of treated virus were used to infect Vero cells in quadruplicate and foci identified based on GFP expression. Infectious titers were normalized (%) to vehicle-treated infected cells set to 100%. Results are presented as means ± SD (four replicates). Results were analyzed (ANOVA) using GraphPad Prism software (v.10); *p* = 0.0965; ns indicates statistically not significant.

To gain insights about the mechanism whereby FLN exerted its antiviral activity against LCMV, we examined which steps of the virus life cycle were affected in the presence of FLN. To examine whether FLN affected a cell entry or post-entry step of the LCMV life cycle, we conducted a time-of-addition experiment using the single-cycle infectious rLCMV∆GPC/ZsG to prevent the confounding factor introduced by multiple rounds of infection (Figure 5a). A549 cells were seeded in a 96-well plate (3.0 × 10^4^ cells/well), and after overnight incubation treated with FLN (50 µM) or VC, starting 2 h prior (−2) or 2 h post (+2) infection (MOI of 1) with rARMΔGPC/ZsG-P2A-NP. ZsG expression levels were determined at 48 hpi and mean values normalized to VC-treated cells that were assigned a value of 100%. RIB and the LCMV cell entry inhibitor F3406 (5 µM) were used as controls. The results showed that FLN exerted a much stronger inhibitory effect on ZsG expression levels when added 2 h prior to infection with rARMΔGPC/ZsG-P2A-NP. The mammarenavirus matrix Z protein has been shown to be the main driving force of budding [51]. To assess whether FLN affected the Z budding activity, we used a published cell-based Z budding assay where the activity of the Gaussia luciferase (Gluc) reporter gene serves as a surrogate of Z budding activity [53]. We transfected HEK293T cells with a plasmid expressing LASV Z-GLuc and treated them with FLN (50 µM) or vehicle control and 48 h later measured levels of GLuc activity associated with VLPs present in CCSs and WCLs (Figure 5b). We used as controls the G2A mutant of the Z protein, which has been shown to be dramatically impaired in its budding activity due to disruption of myristoylation at G2 [56], and treatment with RIB, known not to inhibit Z budding. Budding efficiency was determined by the ratio of VLP-associated GLuc levels (Z_VLP_) and total GLuc levels (Z_VLP_ + Z_WCL_) × 100. FLN had a significant inhibitory effect (50% reduction, **** *p* < 0.0001) on LASV Z budding activity. We also examined whether FLN exerted any virucidal activity on infectious LCMV virions. For this, rLCMV/GFP-P2A-NP (1 × 10^5^ FFU in a volume of 1 ml) was treated with FLN at 1 µM, 50 µM, and 100 µM for 30 min at room temperature. After treatment, samples were diluted 100-fold, resulting in FLN concentrations lacking anti-LCMV activity, and virus infectivity determined by FFA. Treatment with 50 or 100 µM FLN, which caused > 1-log reduction in production of LCMV infectious progeny (Figure 5b), did not significantly affect virion infectivity compared to VC-treated samples (*p* = 0.0965) (Figure 5c).

### 3.6. Effect of FLN on LCMV GPC-Mediated Cell Fusion

**Figure 6:**
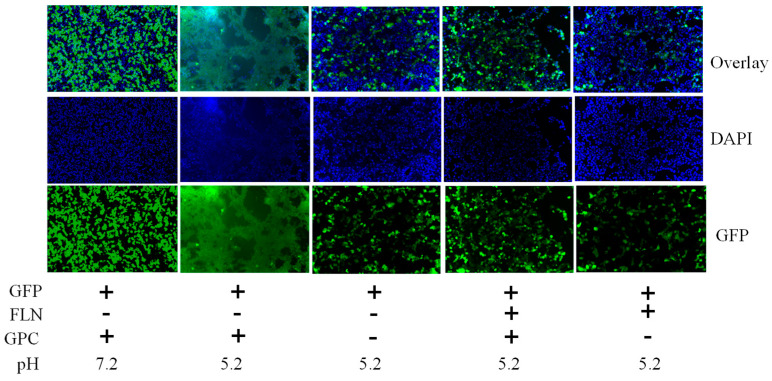
Effect of FLN on GP2-mediated pH-dependent fusion. HEK293T cells were seeded (1.25 × 10^5^ cells/well in 24-well plate). The next day, cells were transfected with pC-LCMV-GPC + pC-GFP + pC-E or pC-GFP + pC-E using Lipofectamine 3000. At 18 h post transfection, the transfection mixture was removed and replaced with fresh media containing VC or drug. After 5 h, cells were exposed to acidified (pH = 5.2) medium for 15 min. The medium was removed and wells washed. Cells were fixed after 2 h, stained with DAPI, and syncytium formation visualized based on GFP expression. The images were taken using a Keyence BZ-X710 at 20× magnification; however, for panels corresponding to GFP (+), FLN (−), and GPC (+) pH (7.2), images were taken at 4× magnification and zoomed out to match images taken at 20×.

Mammarenaviruses enter cells via receptor-mediated endocytosis [57]. Within the endosome’s acidic environment, GP2 facilitates a pH-dependent fusion between viral and cellular membranes, completing the cell entry process and releasing the vRNP into the cytoplasm, where vRNP directs replication and transcription of the viral genome. To investigate whether FLN disrupted the GP2-mediated fusion event, we transfected HEK293T cells with plasmids expressing LCMV GPC (pC-LCMV-GPC) or an empty plasmid (pC-E) as a control, together with a GFP-expressing plasmid (pC-GFP) (Figure 6). At 18 h post-transfection, cells were treated with either VC or FLN (50 µM). After 5 h FLN treatment, cells were subjected to either acidic (pH 5.0) or neutral (pH 7.2) medium for 15 min. Subsequently, cells were returned to regular medium (pH 7.2) for 2 h before being fixed with 4% PFA, stained with DAPI, and examined for syncytium formation based on GFP expression. Cells transfected with LCMV GPC-expressing plasmids and exposed to pH 5 demonstrated robust fusion activity, evidenced by syncytial formation revealed by the GFP expression pattern. In contrast, FLN treatment resulted in the inhibition of GP2-mediated fusion upon exposure to low pH.

### 3.7. Effect of FLN on Multiplication of JUNV

**Figure 7:**
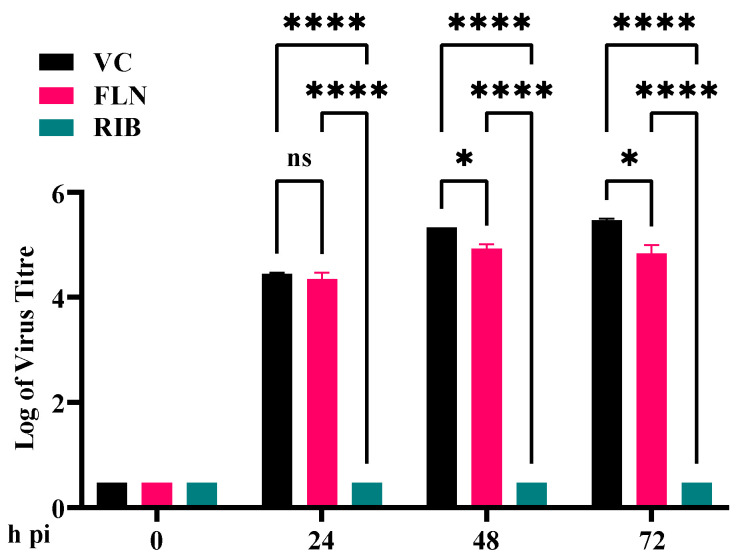
Effect of FLN on JUNV multiplication in Vero cells. Effect of FLN (50 µM) on LCMV multi-step growth kinetics and peak titers. A549 cells were seeded (2.0 × 10^5^ cells/well in 12-well plate) and 16 h later infected with r3JUNVCandid GFP/GFP at an MOI of 0.2. After 90 min adsorption, the virus inoculum was removed and infected cells treated with FLN (50 µM) or RIB (100 µM) as a control. At the indicated time points, post-infection tissue culture supernatants were collected and virus titers determined by FFA using Vero cells. Virus titers shown correspond to the means ± SD. Two-way analysis of variance with Tukey correction for multiple comparisons was implemented for statistical analysis. * *p* < 0.05, **** *p* < 0.0001.

To examine whether FLN exhibited broad-spectrum anti-mammarenavirus activity, we examined its effect on multiplication of the New World mammarenavirus JUNV, which is genetically distantly related to the Old World mammarenaviruses LCMV and LASV. For this experiment, we used a tri-segmented version of the live-attenuated vaccine strain Candid#1 (r3Can) of JUNV expressing the GFP reporter gene (r3Can-GFP). This allowed us to avoid the need for BSL4 biocontainment required for the use of live forms of pathogenic strains of JUNV. FLN (50 µM) inhibited multiplication of r3Can-GFP to levels similar to what we observed with LCMV (Figure 7) (>1-log reduction in virus peak titers compared to VC-treated and infected cells, *p* = 0.0466).

### 3.8. Inhibitory Effect of Selected Calcium Channel Inhibitors on LCMV Multiplication in A549 Cells

**Figure 8:**
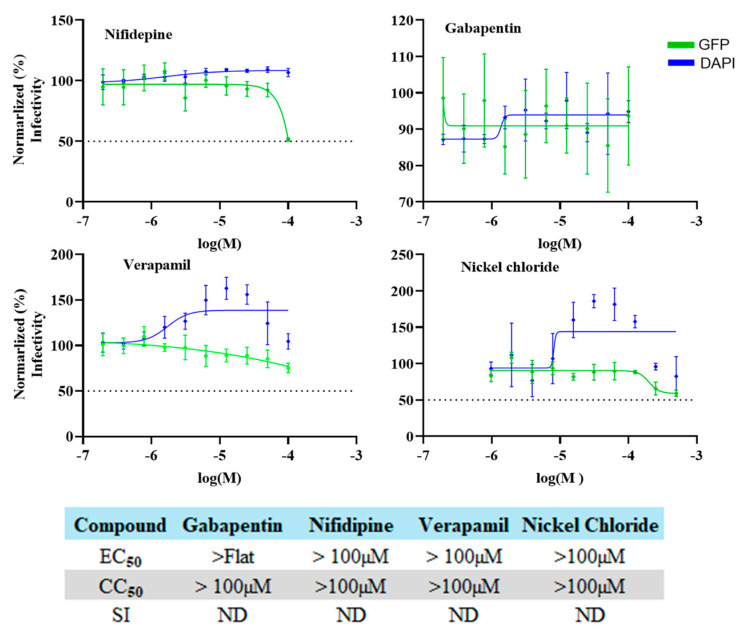
Effect of selected calcium channel blockers on LCMV multiplication in A549 cells. A549 cells were seeded (3.0 × 10^4^ cells/well) in a 96-well plate and 16 h later infected with rLCMV/GFP-P2A-NP at an MOI of 0.03. After 90 min adsorption, the virus inoculum was removed and infected cells treated with 2-fold dilutions of gabapentin (100 µM), nifedipine (100 µM), verapamil (100 µM), and nickel chloride (500 µM) (four replicates per concentration). At 72 h post-infection, cells were fixed with 4% PFA and stained with DAPI. GFP expression levels and DAPI staining were used to determine virus infectivity and cell viability, respectively. Values correspond to the means ± SD of four replicates. Levels of GFP expression and DAPI staining were normalized by assigning a value of 100% to GFP expression levels and DAPI staining of VC-treated and infected cells. Normalized values were used to determine the EC_50_ and CC_50_. ND, not determined.

FLN has been shown to block the activity of low-voltage-activated T-type calcium channels. To assess whether calcium channel blockade was associated with the anti-LCMV activity of FLN, we examined the effect on LCMV multiplication of other selected calcium channel blockers, including the combined L/T-type (verapamil and nickel chloride) and L-type (nifedipine and gabapentin) calcium channel blockers (Figure 8).

### 3.9. Effect of Serum on the Anti-Mammarenaviral Activity of FLN

**Figure 9:**
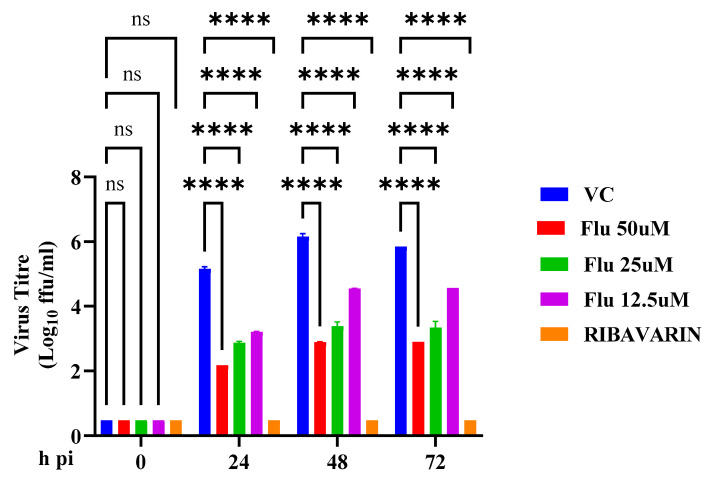
Effect of FLN in the absence of serum on LCMV multi-step growth kinetics and peak titers. A549 cells were seeded (2.0 × 10^5^ cells/well in 12-well plate) and 16 h later infected with LCMV/GFP-P2A-NP at an MOI of 0.03. After 90 min adsorption, the virus inoculum was removed and infected cells treated with different concentrations (50 µM, 25 µM, 12.5 µM) of FLN or RIB (100 µM) in Opti-MEM. At the indicated time points, post-infection tissue culture supernatants were collected and virus titers determined by FFA using Vero cells. The virus titers shown correspond to the means ± SD. Two-way analysis of variance with Dunnett correction for multiple comparisons was implemented for statistical analysis. **** *p* < 0.0001.

More than 99% of FLN can be found bound to plasma proteins [57,58], raising the question of whether the full magnitude of FLN’s anti-mammarenaviral activity was masked in our experiments in the presence of 10% FBS. To address this issue, we examined the effect of FLN on the multi-step growth kinetics of LCMV in the absence of FBS. We found that FLN anti-LCMV activity was 100-fold higher in the absence of FBS (Figure 9) when compared to the assay run in the presence of 10% serum (Figure 4b).

## 4. Discussion

The current standard of care for LF cases is limited, besides supportive care, to an off-label use of the nucleoside analogue RIB, for which efficacy remains controversial [9,12]. Significant efforts are being dedicated to the discovery of antiviral drugs against LASV, which has resulted in the identification of several candidates with potent activity in cell-based infection assays. Notably, the broad-spectrum inhibitor favipiravir (T-705) [59,60] and the LASV GPC-mediated fusion inhibitor ST-193 [61] have shown promising results in animal models of arenaviral hemorrhagic fever (HF) disease, and the ST-193 analogue LHF-535 is in phase I clinical trials [28]. Nevertheless, the development of additional antivirals against LASV can facilitate the implementation of combination therapy against LF, an approach known to counteract the emergence of drug-resistant variants often observed with monotherapy strategies [62]. Limited market opportunities pose significant obstacles for the development and licensing of new drugs against emerging viral diseases endemic to countries with limited resources. Drug-repurposing strategies can significantly reduce the time required to advance a candidate antiviral drug into the clinic by reducing the labor- and resource-intensive efforts involved in preclinical optimization of newly discovered hits in traditional drug-discovery approaches [63,64]. Moreover, information obtained from drug-repurposing efforts can reveal novel insights on virus biology by identifying novel pathways and host cell factors involved in different steps of the virus life cycle, which could uncover new targets and therapeutics. In the present work, we used an in silico docking approach to screen 2015 existing drugs for candidates predicted to bind and functionally affect LASV proteins, as well as two host cell proteins, α-dystroglycan and lysosomal associated membrane protein, known to play critical roles in LASV cell entry. We found that DEX, TAD, MEF, Vit D, and FLN had in silico docking parameters that supported their potential antiviral activity against LASV via targeting components of the LASV vRNP. We evaluated these candidates for their antiviral activity in cell-based MG and cell-based infection assays.

DEX has been reported to have some benefit in the treatment of LF, potentially due to its ability to modulate inflammation-mediated tissue damage associated with LF [55]. TAD, a phosphodiesterase 5 (PDE5) inhibitor, has been found to bind to the viral RNA-dependent RNA polymerase of SARS-CoV-2 [65]. MEF has been suggested to inhibit cell entry of MPOX virus [66]. The role of Vit D in the immune system has come under increasing scrutiny, with research indicating its potential to protect against bacterial and viral invaders via stimulation of production of cytokines, antimicrobial proteins, and pattern recognition receptors, all of which are important for innate immunity. Some studies have suggested that Vit D may offer protection against SARS-CoV-2 infection and be beneficial in the treatment of viral respiratory tract infections [67]. However, we found that DEX, TAD, MEF, and Vit D had minimal inhibitory activities in both LASV and LCMV cell-based MG assays, as well as in LCMV cell-based infection assays. In contrast, the neuroleptic FLN, commonly prescribed in many countries across the world for migraines and vertigo, significantly inhibited LASV and LCMV vRNP activity in cell-based MG assays and LCMV multiplication in cell-based infection assays. Our in silico docking studies predicted that FLN may interact with conserved residues within the EndoN domain of the LASV L protein (Table 2), which may have contributed to the observed inhibitory activity of FLN on the vRNP-directed reporter gene expression in the LASV (~50% inhibition) and LCMV (~75% inhibition) cell-based via targeting of EndoN activity required for transcription of the viral mRNAs. However, whether FLN directly affect the EndoN activity of the L protein remains to be determined. FLN was also predicted to interact with several residues within the C-terminal region of NP involved in NP-Z interaction, which may affect the assembly of infectious progeny. FLN was also predicted to interact with residues K32 and N46 in the Z protein, residues that have been involved in Z-eIF4E interaction. Whether FLN interferes with Z-eIF4E interaction and what the functional consequences may be remain to be determined.

FLN is classified as a Ca^2+^ antagonist that blocks influx of extracellular Ca^2+^, whereas it exerts minimal antagonistic action against dopamine receptors [68]. Ca^2+^ metabolism and signaling having been implicated in different steps of the life cycle of different viruses, including Ebola and Marburg viruses [69], and importantly the mammarenaviruses LASV and JUNV [69]. Notably, FLN has been shown to inhibit hepatitis C virus cell entry by inhibiting HCV E1-mediated pH-dependent fusion [30,68,70], and cell entry inhibitors that target viral components or cellular factors have emerged as an interesting class of inhibitors in HCV [71], as their use in combination with direct acting antivirals targeting virus replication can prevent breakthrough of antiviral-resistant HCV [72]. Our finding that other selected L- and T-type calcium channel blockers did not exhibit anti-LCMV activity suggests that FLN anti-mammarenaviral activity may not be related to its ability to block T-type calcium channels. FLN also exhibits antihistaminic activity via blocking H1 histamine receptors [73,74], and several antihistaminic drugs have been shown to exert antiviral activity [68,75]. However, the strong H1 histamine receptor clemizole does not exhibit anti-mammarenavirus activity [26]. FLN has been shown to bind calmodulin, which could interfere with the role of calmodulin in low-pH-induced fusion of late endosomes and lysosomes [43]. It is plausible that the calmodulin-binding properties of FLN contributed to its inhibitory effect on the GP2-mediated, pH-induced fusion required for mammarenavirus cell entry.

The findings presented in this paper have the limitation of lacking experiments providing evidence supporting the in vivo efficacy of FLN in animal models of mammarenavirus infection. The moderate anti-mammarenavirus activity of FLN in cell-based assays (1-log reduction of virus peak titers), together with its high plasma protein binding (PPB), could pose significant obstacles for FLN exhibiting in vivo efficacy as an antiviral. However, despite its well-established PPB feature, in humans, FLN has been shown to reach sustainable therapeutic levels in plasma in the absence of significant toxicity. Whether FLN therapeutic dosage to treat migraine headaches, FLN main indication in humans, will exhibit also in vivo antiviral efficacy against mammarenaviruses remains to be determined. FLN has not received FDA approval, but its safety is strongly supported by its current use in many countries to treat migraines and vertigo. Moreover, the characterization of novel FLN analogues focused on structural changes in the allylic element and the adjacent arene substituent has resulted in the identification of p-methoxy-FLN with an IC50 ca. 8-fold lower than FLN against HCV, and an SI of 335, which is a 8.8-fold improvement relative to FLN [70]. These findings, together with our results, support the interest in exploring its repurposing as a candidate drug to be used in combination with other antivirals to treat LASV and other human pathogenic mammarenavirus infections.

## Figures and Tables

**Table 1:** Grid box centers and sizes used for molecular docking simulations. Table 1 presents the grid box parameters used for molecular docking simulations of various protein structures. The table includes the Protein Data Bank (PDB) ID for each structure, along with the size of the grid box (in angstroms) in three dimensions (x, y, z) and the coordinates of the center of the grid box (also in angstroms) in three-dimensional space.

PDB ID	Protein	Size			Center		
		x	y	z	x	y	z
2M1S	Z Protein	28	30	23	3.73	0.841	−1.209
3Q7C	Nucleoprotein	15	15	15	−11.41	10.964	−16.37
4ZJF	Glycoprotein 1	15	15	15	−0.07	−4.633	−27.2
5J1N	Polymerase	20	20	20	19.203	1.995	−15.41
5OMI	Glycoprotein 2	22	20	28	1.645	16.212	13.642

**Table 2:** Binding affinities (expressed as Gibbs free energy changes, ΔG). Five drugs were selected by ranking the binding energy (ΔG) of 2015 existing drugs obtained from DrugBank to LASV target proteins. Molecular docking simulations were implemented in four replicates on a Linux platform using AutoDock Vina and associated tools after validation of docking protocols. Binding free energy values (kcal/mol ± SD) were ranked, and dexamethasone (DEX), FLN, tadalafil (TAD), ergocalciferol (Vit D), and mefloquine (MEF) were selected based on the potency of binding activities to the polymerase, Z protein, and nucleoprotein of LASV.

Drug	Polymerase	Z Protein	Nucleoprotein
DEX	−7.40 ± 0.00	−6.63 ± 0.05	−8.20 ± 0.00
FLN	−8.05 ± 0.17	−6.35 ± 0.06	−8.08 ± 0.13
TAD	−7.43 ± 0.10	−7.50 ± 0.00	−7.83 ± 0.05
Vit D	−7.45 ± 0.06	−6.80 ± 0.00	−7.58 ± 0.39
MEF	−8.55 ± 0.06	−6.15 ± 0.24	−7.20 ± 0.00

**Table 3:** Predicted interacting amino acid residues in LASV proteins targeted by selected drugs. Using Discovery Studio, interactions were analyzed to identify specific residues in the L, Z, and NP proteins of LASV that are likely to interact and serve as binding sites for the selected drugs: DEX, FLN, TAD, Vit D, and MEF. These residues represent potential targets for therapeutic intervention, as they suggest where each drug may exert its effect on viral structure or function. The findings are particularly useful in understanding drug–protein interactions and advancing LASV treatment strategies.

Drug	Polymerase	Z Protein	Nucleoprotein
DEX	R24, L48	Y48, K32, H47, L26	L505 A552, H412, H507, V559, F414, R556, Y410
FLN	V87, E102, F104, D66, V105, I50, E51, D89, N63, D119	K74, L31, V60, C64, C44	L505 A552, H412, H507, V559, M508, R556, L554
TAD	V105, F104	K71, S59, C44, C67, N45	L505, A552, C506, M508
Vit D	K44, L48, V105	K74, L75, V60, C64, C67, L71	L505 H507, V559, R556
MEF	V105, C103, E51, R106, D89, E102, D119, F147, I50, S47, F104, K115	N46, K32, P28, F30, Y48	Y410, H507, D504, C506, H412, L554, L505, A552, V559

## Data Availability

Data are contained within the article.

## Supplementary Materials

The following supporting information can be downloaded at https://www.mdpi.com/article/10.3390/v17010117/s1. Table S1: Docking scores of selected existing drugs for LASV proteins; Figure S1: Predicted FLN-interacting amino acid residues in LASV proteins; Figure S2: Predicted FLN-interacting amino acid residues in LASV Z protein.
