# Supplementary material for: Flunarizine as a Candidate for Drug Repurposing Against Human Pathogenic Mammarenaviruses
Source: Viruses. 2025 Jan 16;17(1):117. doi: 10.3390/v17010117 (PMC11768584; doi:10.3390/v17010117)
Supplement: Supplementary file 1 [file viruses-17-00117-s001.zip › Legend of Supplementary Table S1.pdf]

Supplementary Table S1. Docking scores of selected existing drugs for LASV Proteins. Docking scores were obtained from four independent docking simulations for a selection of known drugs. Each row corresponds to a specific compound, listing its individual docking scores (Kcal/mol) across the four simulations, as well as the average of the four independent simulations and their corresponding SD values.
