# Supplementary material for: Flunarizine as a Candidate for Drug Repurposing Against Human Pathogenic Mammarenaviruses
Source: Viruses. 2025 Jan 16;17(1):117. doi: 10.3390/v17010117 (PMC11768584; doi:10.3390/v17010117)
Supplement: Supplementary file 1 [file viruses-17-00117-s001.zip › Supplementary Figure S1.pdf]

Glycoprotein 2	Polymerase	Matrix	Nucleoprotein
Phe309, Met312, Leu313, Phe316, Lys320, Gln324, Ile323, Ile403, Gln406, Met410, Ile411, Met414, Ala407	Val87, Glu102, Phe104, Asp66, Val105, Ile50, Glu51, Asp89, Asn63, Asp119	Lys74, Leu31, Val60, Cys64, Cys44	Leu505 Ala552, His412, His507, Val559, Met508, Arg556, Leu554

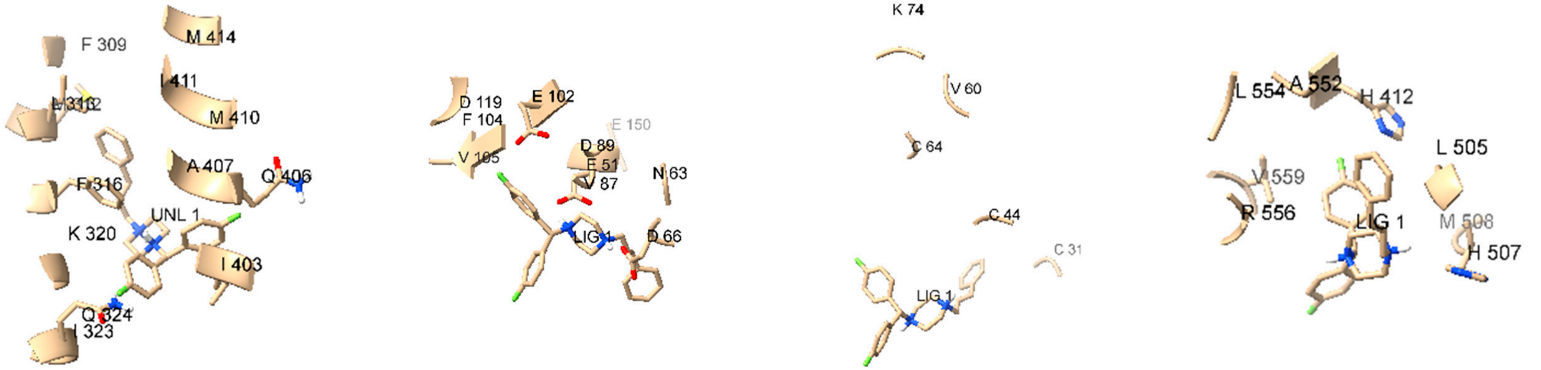

Supplementary Figure S1. Predicted FLN-interacting amino acid residues in LASV proteins. Using Discovery Studio, interactions were analyzed to identify specific residues in GP2, L, Z, and NP proteins of LASV that are likely to serve as binding sites for FLN. The key amino acid residues of LASV proteins predicted to be involved in drug-protein interactions are shown using the one letter code and FLN is indicated as UNL1 in GP2 and as LIG1 in L, Z, and NP Proteins.
