# Supplementary material for: Flunarizine as a Candidate for Drug Repurposing Against Human Pathogenic Mammarenaviruses
Source: Viruses. 2025 Jan 16;17(1):117. doi: 10.3390/v17010117 (PMC11768584; doi:10.3390/v17010117)
Supplement: Supplementary file 1 [file viruses-17-00117-s001.zip › Supplementary Figure S2. JCT.pdf]

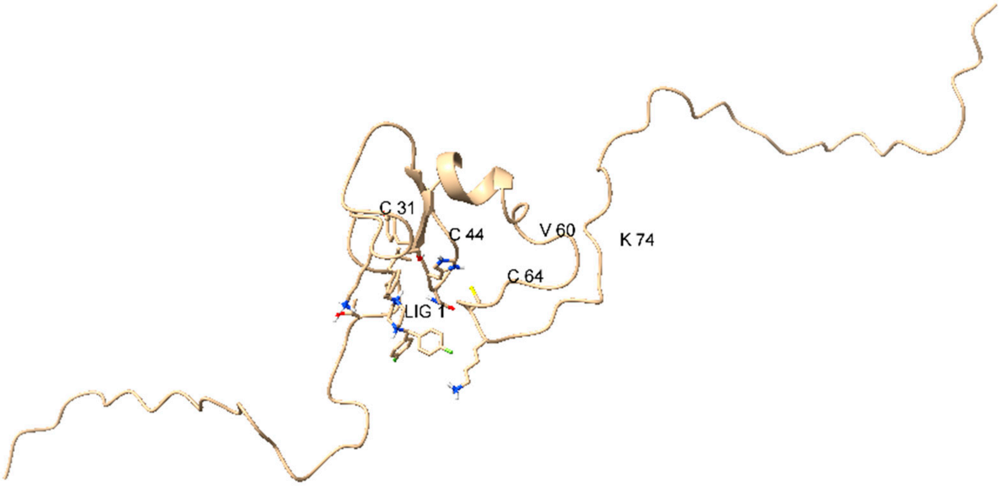

Supplementary Figure S2. Predicted FLN-interacting amino acid residues in LASV Z protein. FLN-Z interaction was analyzed using Discovery Studio to identify specific residues in Z protein that are likely to serve as binding sites for FLN. Amino acid residues predicted to be involved in FLN-Z interaction are shown using the one letter code, and FLN is indicated as LIG1.
